# Anatomical Organization of Multiple Modulatory Inputs in a Rhythmic Motor System

**DOI:** 10.1371/journal.pone.0142956

**Published:** 2015-11-13

**Authors:** Shanna E. Swallie, Alexis M. Monti, Dawn M. Blitz

**Affiliations:** Department of Biology, Miami University, Oxford, OH, United States of America; University of Missouri, UNITED STATES

## Abstract

In rhythmic motor systems, descending projection neuron inputs elicit distinct outputs from their target central pattern generator (CPG) circuits. Projection neuron activity is regulated by sensory inputs and inputs from other regions of the nervous system, relaying information about the current status of an organism. To gain insight into the organization of multiple inputs targeting a projection neuron, we used the identified neuron MCN1 in the stomatogastric nervous system of the crab, *Cancer borealis*. MCN1 originates in the commissural ganglion and projects to the stomatogastric ganglion (STG). MCN1 activity is differentially regulated by multiple inputs including neuroendocrine (POC) and proprioceptive (GPR) neurons, to elicit distinct outputs from CPG circuits in the STG. We asked whether these defined inputs are compact and spatially segregated or dispersed and overlapping relative to their target projection neuron. Immunocytochemical labeling, intracellular dye injection and three-dimensional (3D) confocal microscopy revealed overlap of MCN1 neurites and POC and GPR terminals. The POC neuron terminals form a defined neuroendocrine organ (anterior commissural organ: ACO) that utilizes peptidergic paracrine signaling to act on MCN1. The MCN1 arborization consistently coincided with the ACO structure, despite morphological variation between preparations. Contrary to a previous 2D study, our 3D analysis revealed that GPR axons did not terminate in a compact bundle, but arborized more extensively near MCN1, arguing against sparse connectivity of GPR onto MCN1. Consistent innervation patterns suggest that integration of the sensory GPR and peptidergic POC inputs occur through more distributed and more tightly constrained anatomical interactions with their common modulatory projection neuron target than anticipated.

## Introduction

Central pattern generator (CPG) circuits underlie rhythmic behaviors such as walking, breathing, and chewing in many animals [1–4]. The cellular and synaptic properties of CPGs can be modulated by many sources, including higher order projection neurons, to enable multiple distinct outputs [4–12]. Activity of these projection neurons is regulated by sensory and hormonal input, circuit feedback, and inputs from other regions of the nervous system. These inputs can have effects on projection neurons ranging from rapid cycle by cycle feedback to long term modulatory actions [12–14]. As multiple inputs may act simultaneously to regulate projection neuron activity, their relative anatomical organization could have important functional implications for their integration [13,15–17]. However, little information is available regarding the anatomical organization of multiple inputs to projection neurons, particularly at the level of identified neurons where it should be possible to characterize it [18,19].

**Fig 1:**
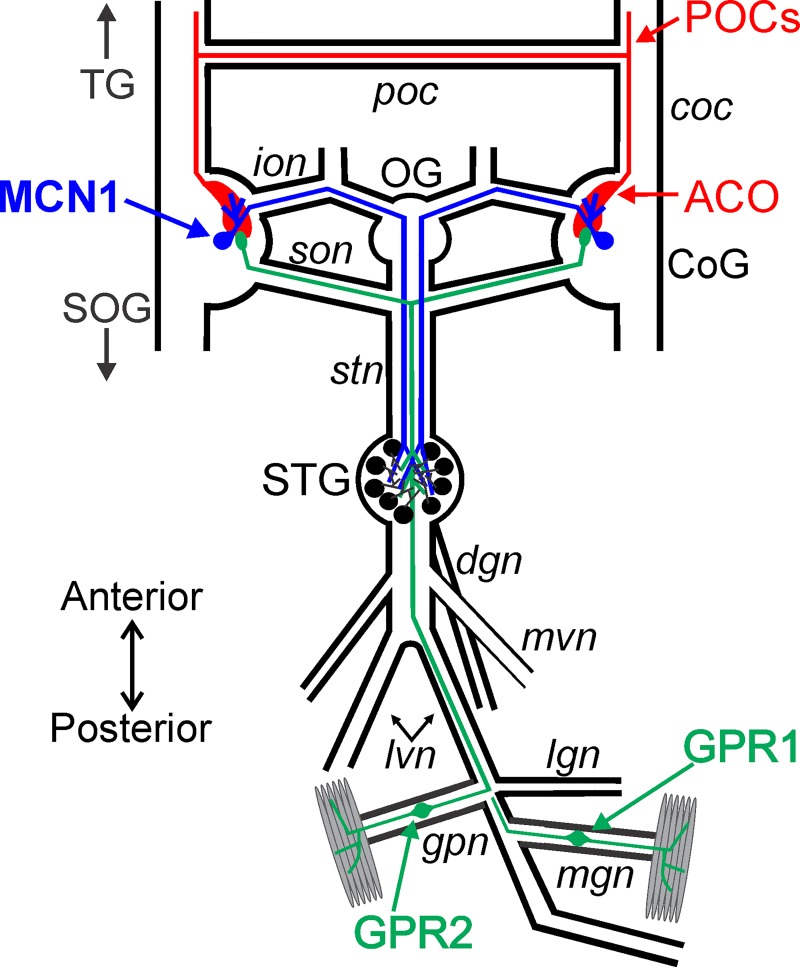
Schematic of the isolated STNS including the projection neuron MCN1, the sensory neurons GPR1/2, and the POC neurons. There is a single MCN1 (blue) cell body in each CoG that projects to the STG. The POC neurons (red) originate outside of the STNS and enter the CoG to terminate as a neuroendocrine organ, the ACO. The somata of the two bilateral pairs of GPR neurons (green) occur in peripheral nerves. These bipolar neurons terminate in muscles and project anteriorly to the STG and continue into the CoGs. Abbreviations: Ganglia- CoG, commissural ganglion; OG, oesophageal ganglion; SOG, supraoesophageal ganglion; STG, stomatogastric ganglion; TG, thoracic ganglion. Neurons- GPR, gastropyloric receptor neuron; MCN1, modulatory commissural neuron 1; POC, post-oesophageal commissure neurons. Nerves: *coc*, circumoesophageal connective; *dgn*, dorsal gastric nerve; *gpn*, gastropyloric nerve; *ion*, inferior oesophageal nerve; *lgn*, lateral gastric nerve; *lvn*, lateral ventricular nerve; *mgn*, medial gastric nerve; *mvn*, medial ventricular nerve; *poc*, post-oesophageal commissure; *son*, superior oesophageal nerve; *stn*, stomatogastric nerve. Other: ACO, anterior commissural organ.

The crustacean stomatogastric nervous system (STNS) enables a cellular level analysis of CPG function and its modulation using identified neurons [12,20]. Projection neurons activate and modulate CPGs, located in the stomatogastric ganglion (STG), which underlie the chewing and filtering of food (Fig 1) [12,21]. The majority of these projection neurons originate in the CoGs (commissural ganglia) with somata located near the surface and neuropil located more centrally [22–24]. The CoGs protrude from the circumoesophageal connectives (*coc*s) connecting the supraoesophageal ganglion (SOG) and the thoracic ganglion (TG) (Fig 1) [22]. Each CoG contains several hundred cells with overlapping distributions of somata projecting to the SOG (~50), to the TG (~100) and to the STG (~20) [23,25,26]. Among the ~20 projection neurons to the STG, a subset have been identified, their influence on STG circuits characterized, and several of their inputs identified [12,27–29]. However, the anatomical organization of inputs to identified projection neurons has not been examined.

The projection neuron MCN1 (modulatory commissural neuron 1) occurs as a single copy within each CoG and projects an axon to the STG where it activates and modulates the pyloric (filtering of food) and gastric mill (chewing) CPG circuits [24,30] (Fig 1). Inputs relaying chemosensory, proprioceptive, mechanosensory, and neuroendocrine signals activate projection neurons including MCN1 in distinct ways, which subsequently results in different outputs from STG circuits [27,31–33]. Two inputs which elicit distinct activation of MCN1 and enable us to ask whether there is segregation or overlap of different inputs, are the post-oesophageal commissure (POC) neurons and the gastropyloric receptor (GPR1 and GPR2) neurons [27,28]. The bundle of approximately 100 POC axons originates outside the STNS, enters the CoG through the anterior *coc* and terminates as a neuroendocrine organ (anterior commissural organ: ACO) [28,34] (Fig 1). Similar to other neuroendocrine systems, the ACO utilizes both endocrine and paracrine signaling [28,34–37]. ACO paracrine actions trigger long lasting MCN1 activity that drives a gastric mill rhythm [28]. The GPR proprioceptor neurons occur as a set of bilaterally paired neurons that originate in the posterior region of the STNS and project anteriorly to the STG and the CoGs (Fig 1). Their actions include excitation of MCN1 resulting in short term activation of a distinct gastric mill rhythm as well as cycle-by-cycle feedback during ongoing motor activity [27,38]. Immunocytochemical analyses in 2D indicate that the four GPR axons enter the CoG and terminate in a compact bundle of presumed synaptic zones. Qualitatively from separate studies, the GPR termination field appears much smaller than the spread of MCN1 neuropil arborization [24,39]. This suggested that contact between MCN1 and GPR would be highly localized. We aimed to determine the extent to which there was segregation of these distinct inputs onto MCN1.

We used confocal microscopy including 3D analysis and took advantage of the modulatory projection neuron, MCN1, occurring as a single copy in each CoG. This enabled examination of anatomical relationships of multiple distinct inputs targeting a projection neuron in a higher order ganglion. We found that despite variable morphology of the ACO neuroendocrine structure, it was consistently coincident with the MCN1 arborization. Further, the two inputs examined here were dispersed and localized to overlapping regions of the MCN1 arborization arguing against spatial segregation of their contacts onto their common target, MCN1.

## Materials and Methods

### Animals

Male crabs (*Cancer borealis*) were obtained from commercial suppliers (Fresh Lobster, Gloucester MA; Ocean Resources, Sedgwick ME) and maintained in filtered, recirculating, artificial seawater holding tanks (10–12°C). Crabs were anesthetized by packing in ice (30–45 min) before dissecting. The foregut of the crab was removed, split along the ventral surface and pinned in a Sylgard 170 (Fisher Scientific) lined glass bowl. The STNS was then dissected off the surface of the foregut under a microscope and pinned in a Sylgard 184 (Fisher Scientific) lined Petri dish [27,40]. The preparation was maintained in chilled *C*. *borealis* saline throughout dissection (~ 4°C). *C*. *borealis* saline contained (in mM): 440 NaCl, 26 MgCl_2_, 13 CaCl_2_, 11 KCl, 10 Trizma base, 5 maleic acid, pH 7.4–7.6.

### Electrophysiology and intracellular dye filling

Extracellular recordings were obtained by placing one wire of a paired electrode along a section of a nerve isolated from the saline by petroleum jelly and the other wire in the main saline compartment. Extracellular recordings of the lateral ventricular nerve (*lvn)*, medial ventricular nerve (*mvn)*, dorsal gastric nerve (*dgn)* and inferior oesophageal nerve (*ion)* were used to monitor the pyloric and gastric mill rhythms and MCN1 activity (Fig 1). MCN1 was identified based on its effects on the pyloric and gastric mill rhythms and its axonal projection through the *ion* [24,30]. Extracellular signals were amplified using A-M Systems 1700 AC amplifiers. Intracellular recordings were obtained using sharp glass microelectrodes (resistance 25–40 MΩ) filled with Alexa 568 in 200 mM KCl (Life Technologies). Signals were amplified using an Axoclamp 900A amplifier (Molecular Devices), digitized at ~5 kHz and recorded using a Micro 1401 data acquisition interface and Spike2 software (Cambridge Electronic Design). Dye was injected using -5 nA current injections for 30–60 minutes. The tissue was continuously superfused with *C*. *borealis* chilled saline (8–11°C).

### Immunocytochemistry

Preparations were fixed overnight in 4% paraformaldehyde (Electron Microscopy Services) in phosphate buffer (Na_2_HPO_4_: 0.1M; NaH_2_PO_4_: 0.1M) and rinsed five times at 1-hour intervals with phosphate buffer containing Triton-X (0.3%; Sigma) (P-triton) before being incubated in primary antibodies. Preparations were again rinsed five times (1-hour intervals) in P-triton prior to incubation with secondary antibody. All antibodies were diluted to appropriate concentrations (Table 1) in P-triton solution. During primary and secondary incubation periods, preparations were placed on a shaker plate. Following secondary antibody incubation, preparations were rinsed five times in phosphate buffer. To prevent compression of CoGs during mounting, the preparation was placed into a chamber fashioned from pieces of coverslip layered with nail polish to create four walls with rubber cement forming the corners of the chamber. Tissue was mounted in 80% glycerol/20mM Na_2_CO_3_ inside the chamber, coverslips were placed on top of the walls of the chamber, and the chamber was sealed with rubber cement.

**Table 1:** 

Antibody	Antigen	Immunogen	Source	Dilution
Anti-serotonin	Serotonin	Serotonin coupled to bovine serum albumin	Immunostar Catalog # 20080 Lot # 1131001 rabbit, polyclonal, RRID:AB_572263	1:500
Anti-substance P	CabTRP Ia	C-terminal BSA-linked substance P	Accurate Chemical and Scientific Catalog # YMC1021 No. NC1/34 HL rat, monoclonal, RRID:AB_2314055	1:300

### Antibody characterization

A rabbit polyclonal serotonin antibody (Immunostar; 1:500 for 24 hours) (Table 1) and goat anti-rabbit Alexa Fluor 488 secondary antibody (Life Technologies; 1:300 for 16–18 hours) were used to label the serotonergic GPR neurons [39]. Specificity of the serotonin antibody was demonstrated through preabsorption with 10^−6^ M serotonin coupled to bovine serum albumin (BSA), which abolished labeling in crustacean nervous systems [41]. The distribution of serotonin-immunoreactivity in this study was similar to the previously characterized distribution in the STNS [39]. The GPR neurons are not the only serotonin-IR neurons arborizing within the CoGs, however, they are the only serotonin-IR axons within the *stn* and *son*s (Fig 1) [39]. In a subset of preparations, we obtained tiled images to reveal the entire region from the *stn* through the *son*s to the CoGs (Fig 1), and verified that the axon bundle entering the CoGs from the *son*s were the GPR axons (n = 3) [39]. Only labeled structures that could clearly be traced from the GPR axons entering through the *son*s were included in analysis.

A monoclonal antibody generated against substance P (Accurate Chemical and Scientific Corporation; 1:300 for 72 hours) (Table 1) was used with goat anti-rat Alexa Fluor 488 or 568 (dilution of 1:300 for 16–18 hours; Life Technologies) secondary antibody to label the POC axons and their terminals (ACO). The substance P antibody specifically recognizes the conserved C-terminal of substance P and related peptides [42–44]. This includes specificity for the endogenous crab peptide, *Cancer borealis* tachykinin related peptide Ia (CabTRP Ia). Labeling in the STNS is blocked by preabsorption with substance P (10^−7^ M) and with native CabTRP Ia (10^−4^ M) [43,44]. The distribution of CabTRP Ia immunoreactivity in this study was similar to previous studies [28,34,43]. As the native *C*. *borealis* peptide recognized by the monoclonal antibody generated against substance P has been identified, we will refer to labeling with this antibody as CabTRP Ia-immunoreactivity (CabTRP Ia-IR). MCN1 also contains CabTRP Ia and is labeled with the Substance P antibody. However, the MCN1 label is typically weak while the ACO label is intense [28, 34]. In order to prevent saturation of the ACO label, it was necessary to decrease the illumination intensity much below that necessary to visualize CabTRP-IR in MCN1. In preparations in which MCN1 was labeled with Alexa dye the Alexa labeled MCN1 soma, axon, and neurites which did not overlap with the ACO structure were not visible in the CabTRP Ia only channel (e.g., Fig 6A and 6D). This was verified in each preparation in which double labeling was performed. Additionally, the ACO is a flocculent structure [34] (e.g., Figs 5–7), distinct from the more typical neuronal branch structure of MCN1 neurites. Thus, the distinct morphologies and intensity of CabTPR Ia-IR of MCN1 and the ACO enabled identification of CabTRP Ia-IR as ACO processes, distinct from MCN1 neurites, using appropriate confocal microscope settings.

### Confocal microscopy and image processing

The tissue was scanned using a Zeiss 710 laser scanning confocal microscope using dry objectives (10x, NA = 0.30; 20x, NA = 0.80; 40x, NA = 0.75). Images were obtained on a 1,024 x 1,024 grid field of view. Differential interference contrast (DIC) was used to take single slice images at various depths through the ganglion to view the outline of the tissue. Zen software (ZEN 2009, Zeiss) was used for image processing including contrast enhancements, maximum intensity projections, 3D analysis, volume rendering and depth coding. For some images, small uniform increases in brightness and contrast were applied with Corel Photo-paint (Corel Corporation) to brighten images (Figs 6D–6F, 7, 10 and 11). To optimally visualize 3D relationships within the volume of an image, maximum projections or volume renderings were used. Specifically, maximum intensity projection refers to 2D images in which each pixel contained the maximum intensity in that pixel location when compared across all slices within a z-stack. For volume rendering, 3D images were calculated with a transparent effect and rendered as 2D images using Zen software. Depth coding applied a pseudocolor code based on the z plane of each optical slice within a z-stack (Zen software). Images in figures are single optical slices, maximum projections or volume renderings of an image stack as indicated.

### Figures and data analysis

**Fig 2:**
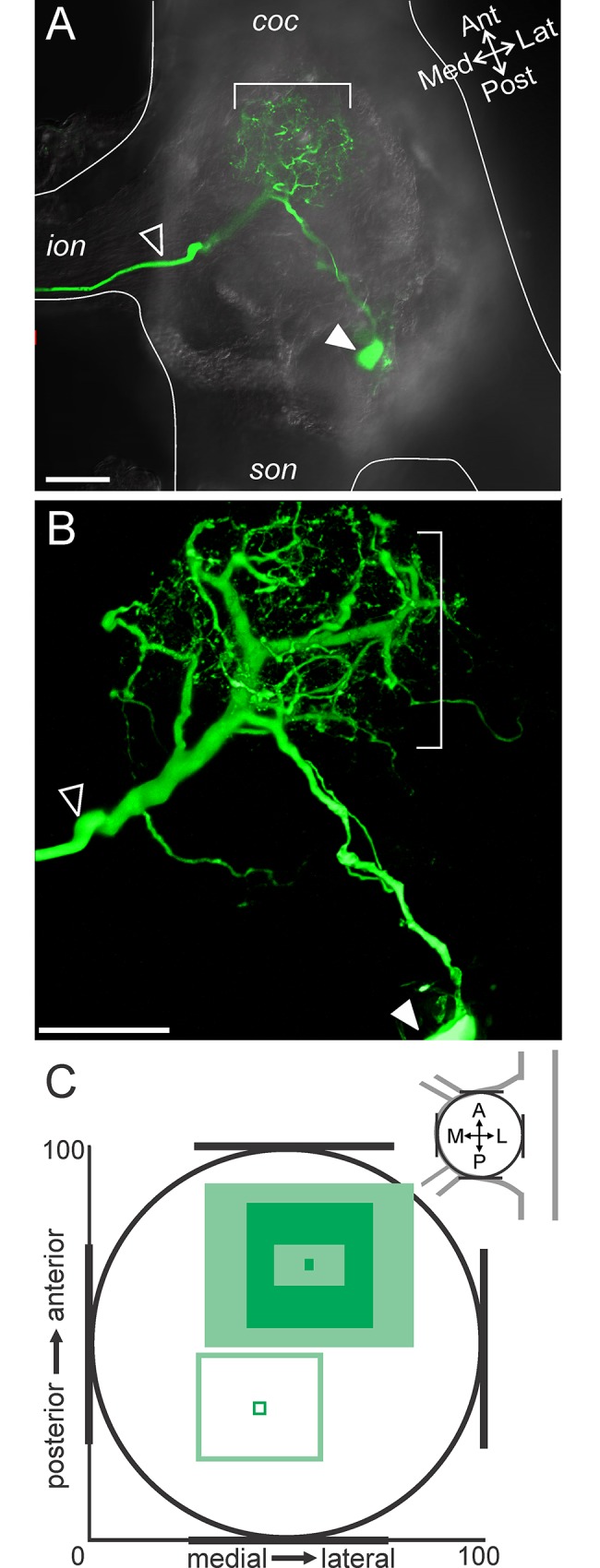
MCN1 neurites arborize in the anterior CoG. (A) A single optical slice includes a portion of MCN1 (intracellular fill with Alexa 568; green) and DIC optics to view the outline of the tissue. The MCN1 neurites are located in the anterior region of the CoG (bracket), while in this example, the soma is located more posteriorly (filled arrow) and the axon leaves the CoG through the *ion* (open arrow). (B) A higher magnification volume rendering of a z-stack (249 optical slices, 1.0 μm interval) of the MCN1 fill from (A) reveals the full extent of the MCN1 arborization within the anterior CoG. Arrows as in (A). (C) Average location of the MCN1 soma (open square) and MCN1 neurites (filled squares) in the x-y plane of the CoG are plotted. The soma was located in the posterior CoG while the neurites were in the anterior region. The neurite location is reported as the average (dark filled center box) and standard deviation (lighter center box) of the center of the arborization and the average (dark filled outer box) and standard deviation (lighter outer box) of the spread of the arborization as measured vertically and horizontally from center across preparations. Scale bars: 100 μm. *coc*, circumoesophageal connective; *ion*, inferior oesophageal nerve; *son*, superior oesophageal nerve.

To quantify locations in the anteroposterior and mediolateral dimensions, both DIC and fluorescence signals were collected at multiple dorsoventral planes. For each ganglion, a single optical slice of the CoG at the depth at which the diameter of the ganglion was greatest was used to identify borders of the ganglion. Specifically, tangent lines along a standardized circular shape were aligned to the anterior, posterior, and medial edges of the CoG, with the circle oriented such that the lateral tangent line aligned with the axon tract of the *coc* (Fig 2, inset) (Corel Draw, Corel Corporation). In each preparation, this alignment was then maintained across single optical slices above and below the widest diameter slice to quantify structure locations in the anteroposterior and mediolateral axes. This allowed us to collapse the analysis into a two-dimensional plot despite differences in the diameter of the ganglion throughout the dorsoventral axis. To normalize the data and eliminate differences due to inter-preparation variability in CoG size, measurements were scaled such that medial was designated as 0, lateral as 100, posterior as 0, and anterior as 100 in the plane in which the CoG diameter was greatest (Fig 2). All analyses of structures throughout the depth of a ganglion were performed on a single z-stack from a continuous confocal session to ensure alignment of optical slices. Using a grid overlaid on the circle in each relevant image (VistaMetrix software; SkillCrest) locations of the MCN1 soma, MCN1 neurites, the ACO, and the GPR axon bundle were quantified. For larger structures such as the MCN1 neuropil and the ACO, both the center and the margins in the anteroposterior and mediolateral dimensions were determined. For the MCN1 soma and the GPR axon bundle, only the center of the structure was determined.

The most ventral surface of each CoG was set as 0 and the most dorsal surface was set as 100 to normalize data across preparations. The center of the MCN1 soma, the dorsal and ventral margins of the MCN1 neuropilar arborization, the center of the ACO, the POC axons, the GPR axon bundle, and the point at which the GPR axons defasciculated were quantified relative to the ventral and dorsal margins. The center of the ACO was identified between the dorsal and ventral margins of the main body of the ACO, excluding the POC axons. Data are presented as average and standard deviation. Figures were constructed using Zen, Corel Draw and Sigma Plot software (v12.5; Systat Software Incorporated).

## Results

### MCN1 localization in the CoG

After obtaining an intracellular recording, MCN1 was identified based on its axonal projection through the *ion*, and its effects on the pyloric and gastric mill circuits [24,30,45]. Once identified as MCN1, the neuron was filled with Alexa Fluor 568.

To examine the location of the MCN1 soma and neuropilar arborization within the anteroposterior and mediolateral dimensions, we used DIC optics to visualize the outline of the CoG paired with a fluorescently labeled MCN1 (see Materials and Methods). Similar to Coleman and Nusbaum [1994], we found that the MCN1 neurites were located in the anterior region of the CoG (Fig 2A). A single primary neurite projected from the MCN1 soma and branched into an axon and 1 (n = 1/10) or 2 (n = 9/10) sub-primary neurites. Fine higher order branches arose from the primary and sub-primary branches (Fig 2B; n = 10/10). The MCN1 soma had a diameter of 46.6 ± 9.0 μm (n = 8) and varied in location across preparations. Although the MCN1 soma was visible in all 10 intracellular fills, in two preparations the cell body appeared to have ripped during processing and these were excluded from diameter measurements. We quantified the MCN1 soma and neurite locations (see Materials and Methods and Fig 2C) and found that the MCN1 soma occurred most often in the posterior half of the CoG (n = 8/10), but with a large standard deviation in the anteroposterior and mediolateral axes (Fig 2C) (n = 10). The neurites, in contrast, were consistently located in the anterior region of the CoG but also varied in the extent of the anterior portion they occupied and in their mediolateral spread indicated by the standard deviation (Fig 2C) (n = 10). Despite variability of both soma location and neuropil spread within the CoG, their relative positions were fairly consistent in the anteroposterior axis with the MCN1 soma most often located posterior to the neuropil arborization (n = 9/10) (Fig 2C).

**Fig 3:**
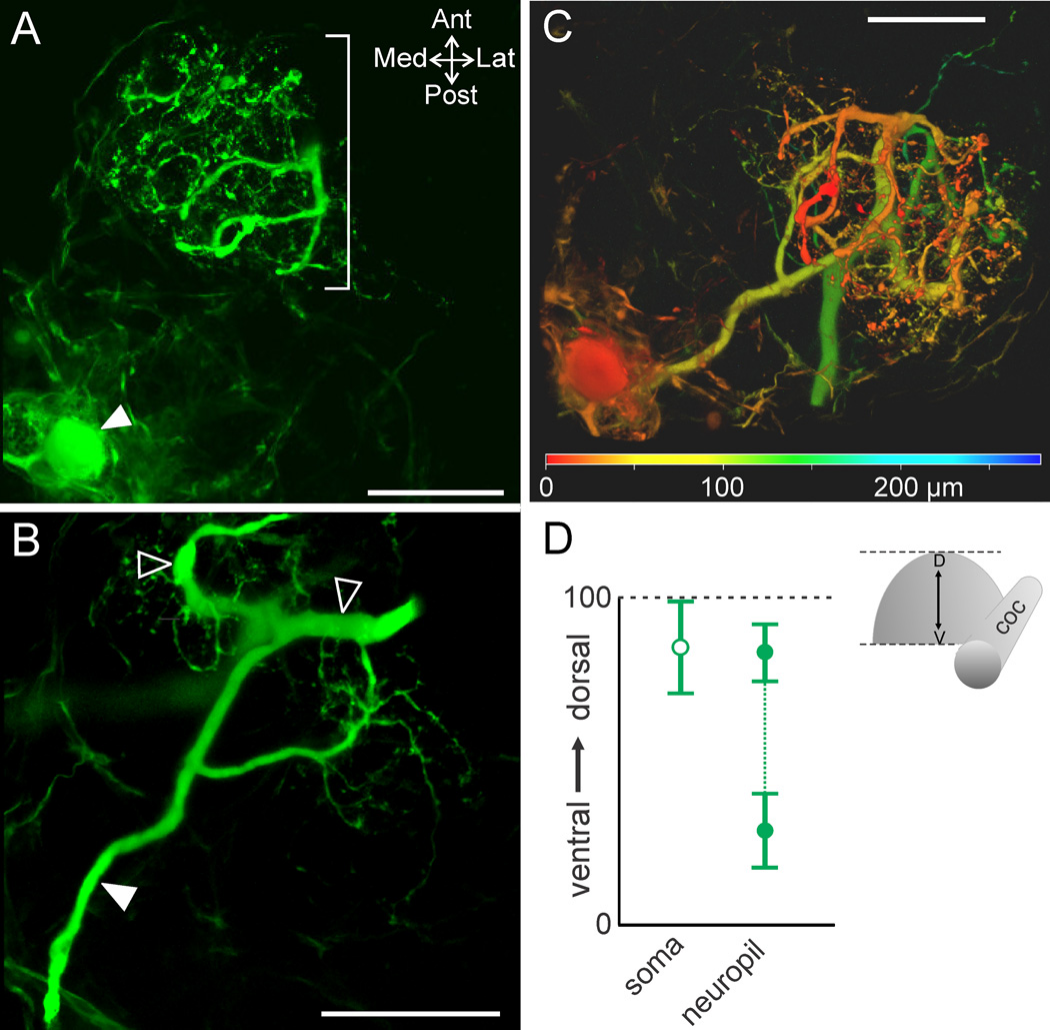
MCN1 neurite size differs across the dorsoventral axis. (A) Volume rendering of MCN1 soma (filled arrow) and smaller neurites (bracket) in the most dorsal portion of the neurite arborization (volume rendering of z-stack: 46 optical slices, 1.0 μm interval). (B) Thicker neurite branches (open arrowheads) occur near the center of the depth of the arborization (volume rendering of z-stack: 49 optical slices, 1.0 μm interval). Filled arrow indicates axon. (C) Volume rendering of a z-stack (281 slices, 1.0 μm interval) is shown with depth coding applied to demonstrate the distribution of MCN1 soma and neurite branches throughout its full depth. The color scale at the bottom of the image indicates the depth from dorsal (red) to ventral (green/blue). The MCN1 soma and smaller neurite branches are more dorsal than the thicker neurite branches. Scale bars: 100 μm. (D) Average (± S.D.) location of the MCN1 soma (open circle) in the dorsoventral plane and the dorsal- and ventral-most extent of the neuropil (closed circles) are plotted (dorsoventral axis normalized from 0 to 100; see Methods).

Relative to the dorsoventral extent of the STG (~65 μm) [46], the CoGs had a larger dorsoventral extent (309.5 ± 46.4 μm; n = 30) that could potentially permit segregation of branching patterns across the dorsoventral dimension. Thus, we also examined the distribution of the MCN1 neuropil arborization throughout the dorsoventral dimension of the CoG. The neurites extended throughout the CoG depth. The most ventral neurite branches were located at an average of 28.8 ± 11.3 (range 0–100, see Materials and Methods) while the most dorsal were located at 83.1 ± 8.8 (n = 10). The most dorsal (Fig 3A) and ventral branches tended to be smaller in size while the branches in the middle of this range were larger diameter in 9 out of 10 preparations (Fig 3B). The MCN1 soma was typically located closer to the dorsal surface than the neurites or located near the dorsal limit of the neurites (82.2 ± 14.2, n = 10) (Fig 3D). To highlight these distinctions while visualizing the entire MCN1 arborization, depth coding was applied to a maximum projection of a MCN1 z-stack. This illustrates that the soma was located in the dorsal region (red) while MCN1 neurite branches spanned from dorsal (red) to ventral (green/blue) regions of the CoG (Fig 3C).

### ACO/POC arborization in the anterior CoG

**Fig 4:**
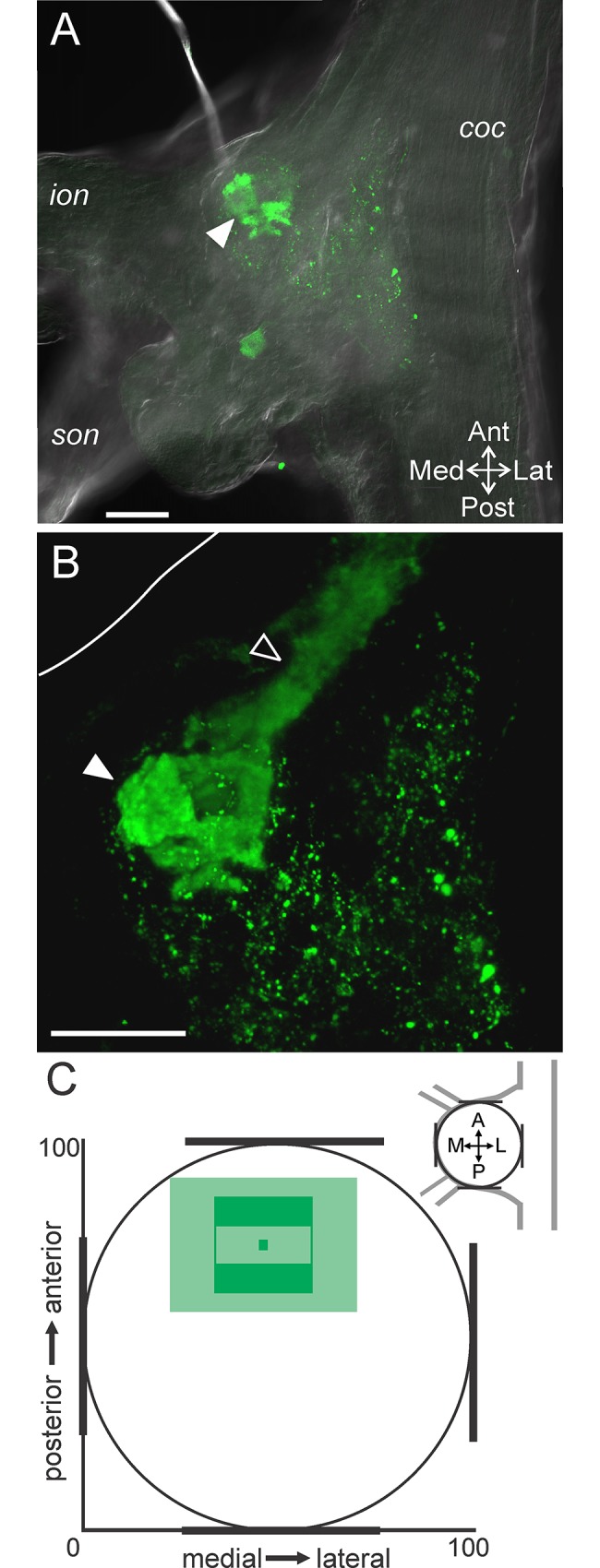
The ACO consistently occurs in the anterior region of the CoG. (A) A single optical slice including fluorescence signal (green: CabTRP Ia-IR) and DIC optics demonstrates that the ACO (filled arrowhead) was located in the anterior region of the CoG. (B) A volume rendering (170 slices, 1.0 μm interval) of the same ganglion as in (A) illustrates that the POC axons (open arrowhead) entered the CoG from the anterior *coc* and terminated as the large ACO structure (filled arrowhead). White line marks the edge of the CoG visible in (A). Scale bars: 100 μm. (C) The average location of the ACO indicates a consistent occurrence in the anterior portion but a variable location across the mediolateral axis. The ACO location is reported as the average (dark green center box) and standard deviation (lighter green center box) of the center of the ACO and the average (dark green filled outer box) and standard deviation (lighter green outer box) of the spread of the arborization as measured vertically and horizontally from center. *coc*, circumoesophageal connective; *ion*, inferior oesophageal nerve; *son*, superior oesophageal nerve.

The CabTRP Ia immunoreactive axons entering the CoG through the anterior *coc* arise from neurons named after their projection through the post-oesophageal commissure (*poc*: POC neurons), while their terminals form a neuroendocrine organ called the anterior commissural organ (ACO) based on its location within the CoG [28,34]. We maintain this nomenclature, referring to the axons as POC and the axon terminals that comprise the neuroendocrine organ as ACO. To determine the consistency of the ACO location within the CoG, we quantified its location relative to the margins of the CoG. A single optical slice combining DIC optics with CabTRP Ia-IR illustrates that the ACO was located close to the anterior CoG boundary (Fig 4). This optical slice contained the widest portion of the ACO. A volume rendering of a z-stack reveals that the POC axons entered the CoG from the anterior *coc* and terminated as the ACO, which arborized entirely within the anterior CoG (Fig 4B). In 14/14 preparations, the ACO was consistently contained in the anterior half of the CoG, while it spread across a large extent of the mediolateral CoG axis (Fig 4C).

**Fig 5:**
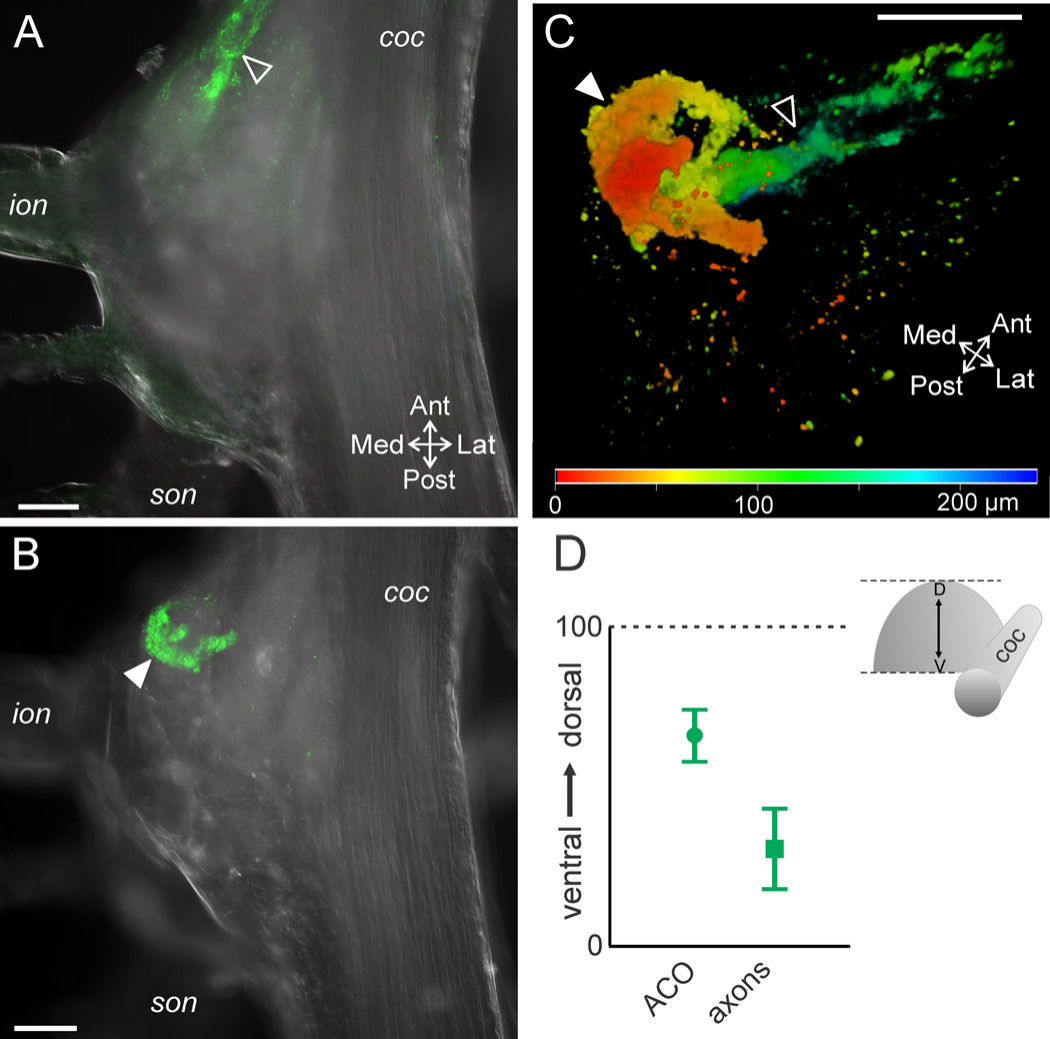
The POC axons and the ACO neuroendocrine organ differ in their dorsoventral locations. Single optical slices with CabTRP Ia-IR (green) and DIC optics illustrate that (A) the POC axons (open arrowhead) entered the CoG near the ventral surface (70 μm from the ventral CoG surface), while (B) the ACO (filled arrowhead) occurred more dorsally (184 μm from ventral CoG surface, 94 μm from the dorsal CoG surface) than the POC axons. (C) Depth coding (red = dorsal, green/blue = ventral) applied to a volume rendering of a z-stack of the same CoG as above highlights that the POC axons (open arrowhead) are located more ventral than their termination as the ACO (filled arrowhead) (201 slices, 1.2 μm interval). (D) Average (± S.D.) locations of the center of the ACO structure (circle) and the POC axons (square) in the dorsoventral plane are plotted. Scale bars: 100 μm. *coc*, circumoesophageal connective; *ion*, inferior oesophageal nerve; *son*, superior oesophageal nerve.

The ACO and the POC axons were distributed beyond the margins of a single optical slice (Fig 4A), thus we examined the ACO distribution through the depth of the CoG. As with our examination of MCN1, we identified the most ventral (0) and dorsal (100) CoG limits and then the dorsoventral extent of the POC axons and ACO relative to these CoG boundaries. The POC axons entered the CoG on the ventral side (32.2 ± 13.1, n = 14) (Fig 5A). They then turned and projected dorsally as the axons branched into the ACO (Fig 5B). The center of the ACO consistently occurred closer to the dorsal surface (65.6 ± 8.5; n = 14) than the POC axons (Fig 5D). This separation of the POC axons (green) and the ACO (red-yellow) in the dorsoventral axis is evident in a volume rendered image to which depth coding was applied (Fig 5C).

### ACO/MCN1 relationship

**Fig 6:**
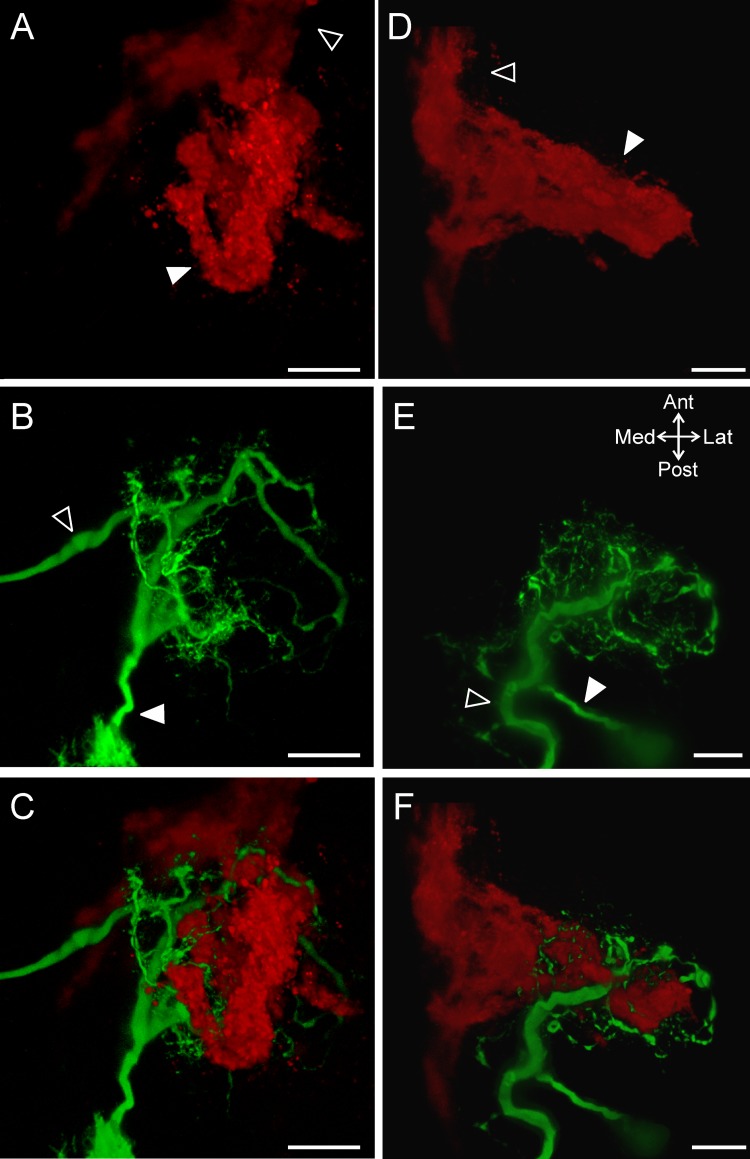
The ACO and MCN1 morphologies are similar within preparations despite variability across preparations. Volume renderings of z-stacks demonstrate the arborizations of the ACO (A, D) and MCN1 (B, E) and their organization relative to each other (C, F) in two preparations. Both in an example of a round-shaped ACO (A-C) and an elongated ACO (D-F), the MCN1 and ACO arborizations share a similar morphology. Images consist of CabTRP Ia-IR (ACO: red) and intracellular fills of MCN1 with Alexa 568 (green). A-C: 231 slices, 1.0 μm interval; D-F: 246 slices, 1.0 μm interval. Open arrows indicate the MCN1 axon (B, E) and POC (A, D) axon bundle while the filled arrows point to the MCN1 primary neurite (B, E) and the ACO (A, D). Scale bars: 50 μm.

Although the majority of ACOs (n = 13/18) were generally round in shape (ex: Figs 4 and 6A), a subset of ACOs (n = 5/18) had an elongated shape as they projected into the anterior region of the CoG (ex: Fig 6D). The MCN1 neurite arborization closely resembled the structure of the ACO, whether it had a round morphology (Fig 6B and 6C; n = 2/2) or an elongated morphology (Fig 6E and 6F; n = 2/2).

**Fig 7:**
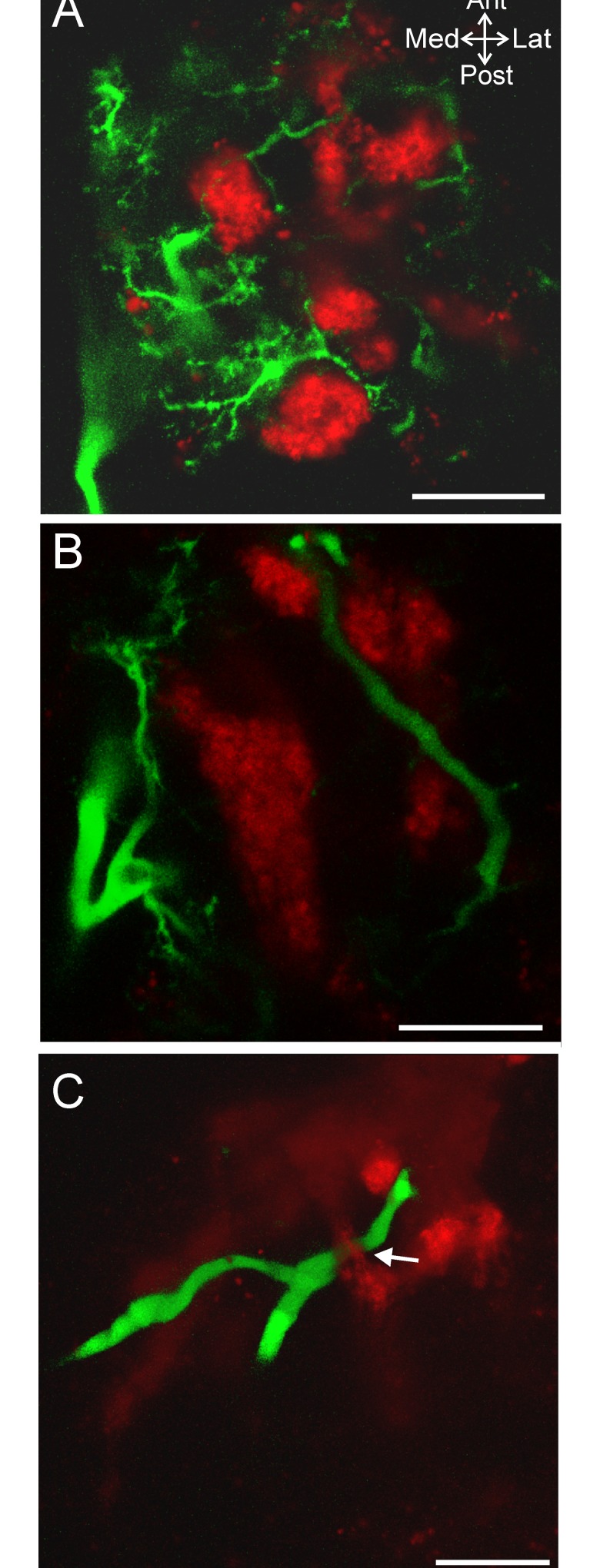
The ACO and MCN1 neurites are intertwined with each other throughout the depth of the CoG. (A) In a dorsal ACO region (CabTRP Ia-IR: red), small MCN1 neurites (Alexa 568 fill: green) occurred in close proximity to the ACO including wrapping around segments of the ACO and passing through gaps within the ACO structure (volume rendering of z-stack, 5 slices, 1.0 μm interval). (B) In a region of the same ACO near the middle of the CoG depth, thicker MCN1 neurite branches wrapped around the ACO (volume rendering of z-stack, 6 slices, 1.0 μm interval). (C) MCN1 and the ACO also were intertwined in the ventral CoG as illustrated in this example in which a MCN1 neurite branch passed through a loop (arrow) formed in the ACO structure (volume rendering of z-stack, 119 slices, 1.0 μm interval). Scale bars: 50 μm.

We found that the MCN1 neurites wrapped around the ACO structure including projecting through gaps. Although in this study we did not label hemolymph vessels, the majority of gaps in the ACO indicate the presence of hemolymph lacunae [34]. The weaving of MCN1 neurites through the ACO structure is more apparent when looking at smaller sections of tissue with less overlap in the dorsoventral dimension. In subsets of z-stacks MCN1 neurites can be seen wrapping around and through gaps in the ACO structure (Fig 7). This was true at three different depths from dorsal (Fig 7A) to ventral (Fig 7C) in the example shown. In 8/18 examples, a branch from the POC axons split from the main bundle and projected into the CoG separate from the main ACO structure. This branch either rejoined the axons or ACO (n = 4/8) or did not rejoin and instead formed a fingerlike projection (n = 4/8). These separate branches tended to be located more ventrally than the main ACO structure. However, in preparations in which MCN1 was also labeled, the separate ACO branches were always located near a ventral section of MCN1 neurite branches (n = 4/4). In an example in which the branch formed a loop, a single large MCN1 neurite branch passed through the loop and continued dorsally (Fig 7C). Thus, irrespective of the ACO shape or extent of the CoG throughout which the ACO branched, there were consistently MCN1 neurites intertwined with the ACO structure. Overall, these data demonstrated extensive overlap of the ACO structure and the MCN1 neuropil arborization.

### GPR projection into the anterodorsal CoG

The four GPR neurons project from the posterior region of the STNS and enter the CoGs through the bilateral *son*s (Fig 1) [39]. The four axons travel closely associated with each other and were thought to terminate in a compact bundle in the anterior CoG. This suggested connectivity between GPR and CoG targets would be limited to a small region. We were thus interested in determining the organization of the GPR neurons in more detail within the 3D structure of the CoG.

**Fig 8:**
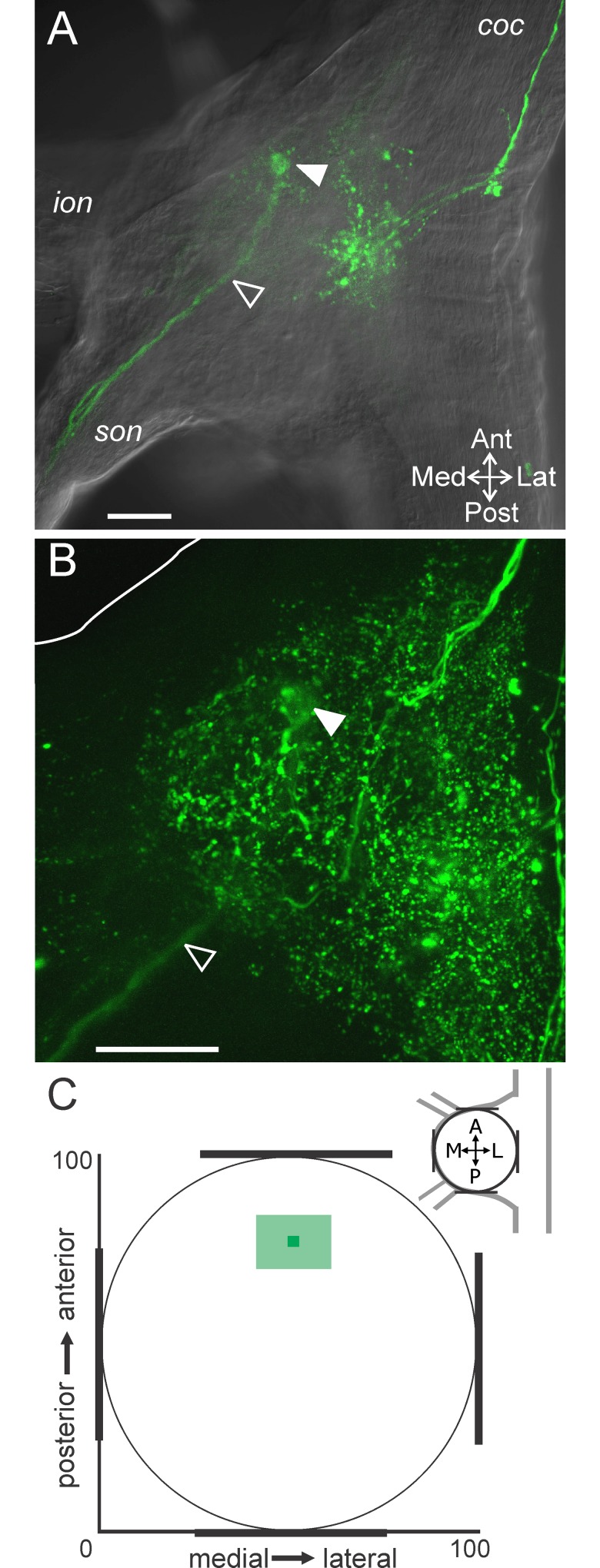
The GPR axon bundle projects into the anterior CoG region. (A) The GPR axon bundle (serotonin-IR; open arrowhead) entered the ganglion through the *son* and projected into the anterior region of the CoG where the axons appeared to terminate in a compact bundle on this plane (filled arrowhead). The tissue was visualized with DIC optics (single optical slice). (B) A maximum intensity projection of the same CoG as in (A) reveals additional serotonin-IR while the GPR axon bundle is still evident projecting into the anterior region and appearing to terminate as a bundle (filled arrowhead) (268 slices, 0.8 μm steps). The white line indicates the outline of the tissue in the anteromedial region. Scale bars: 100 μm. (C) A plot of the average (± S.D.) location of the apparent GPR axon bundle termination indicates a consistent occurrence in the anterior CoG. *coc*, circumoesophageal connective; *ion*, inferior oesophageal nerve; *son*, superior oesophageal nerve.

A single optical slice visualizing the CoG with DIC optics and the GPR axons with serotonin immunoreactivity (serotonin-IR) at the level of the *son* illustrates that the GPR axons entered through the *son* and projected into the anterior region of the CoG where they appeared to terminate as a compact bundle (Fig 8A). In a maximum projection through the depth of the CoG, in addition to the bundle of GPR axons, serotonin-IR neuropil is visible throughout the CoG (Fig 8B). 5HT-IR includes additional structures besides the GPR axons. Thus, all GPR labeling was always traced from axons entering through the *son* as GPR axons are the only 5HT-IR axons in this nerve [39]. We traced the GPR axons from their entry into the CoG to their farthest projection in the anteroposterior and mediolateral dimensions, and found the farthest projection to be consistently located in the anterior CoG, approximately at the midpoint along the mediolateral axis (Fig 8C) (n = 15).

### GPR/MCN1 relationship

**Fig 9:**
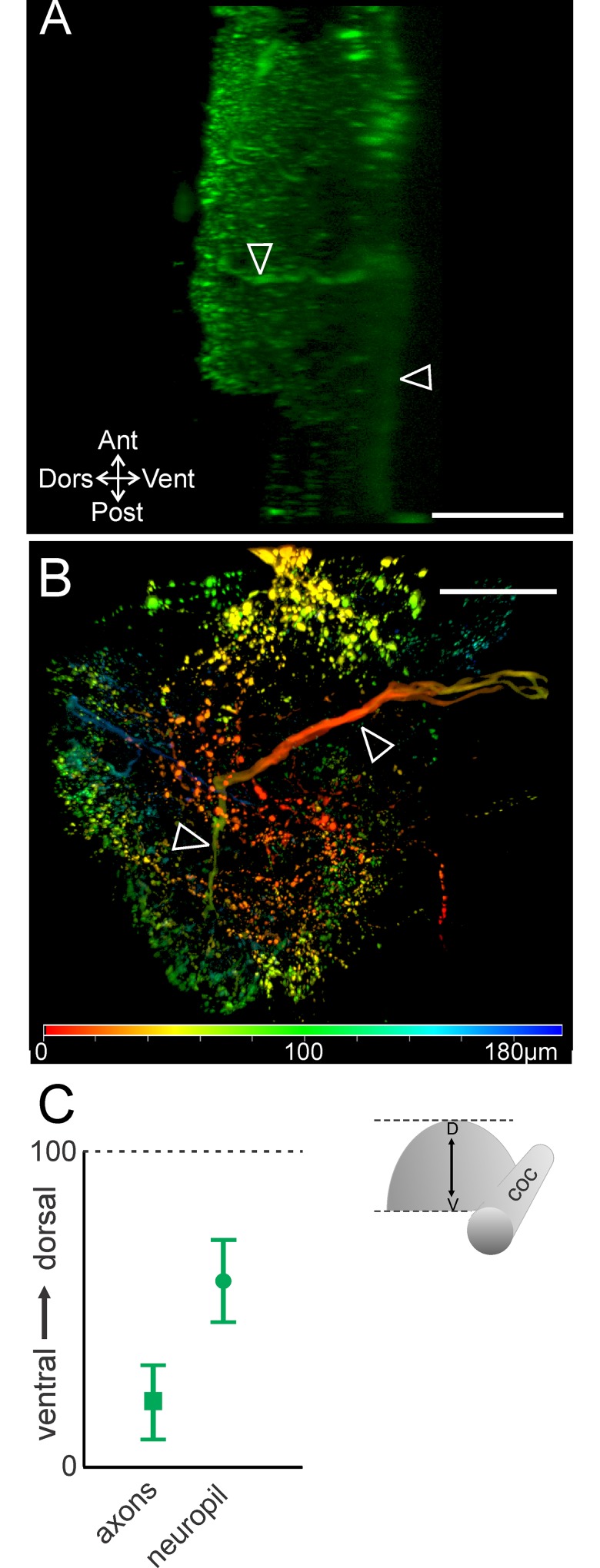
GPR axons enter the CoG ventrally and turn to project dorsally. (A) A side view of a z-stack (268 slices, 0.8 μm interval) reveals that the GPR axon bundle (open arrowheads) entered the CoG at the ventral surface and then turned to project dorsally. (B) The ventral to dorsal axonal trajectory of GPR is also illustrated by applying depth coding to a z-stack (volume-rendering, 191 slices, 1.0 μm interval). This preparation was mounted with the ventral surface toward the coverslip which increased the clarity of the GPR axons on the ventral surface. The GPR axon bundle (open arrowheads) entered the CoG ventrally (red) and then continued into the CoG by turning and projecting into the dorsal region (green). (C) Average (± S.D.) locations of the GPR axons (square) and the point at which the GPR axons defasciculate (circle) in the dorsoventral plane are plotted. (A) and (B) are from different preparations. Scale bars: 100 μm.

Given the three dimensional structure of MCN1, we were interested to determine the plane of the GPR axons relative to MCN1. Using DIC optics to determine the CoG dorsoventral margins as above, we found that the GPR axon bundle at its farthest anterior projection was near the ventral surface (20.5 ± 11.8) (n = 15) (Fig 9C).

Previous studies had used two-dimensional views of the CoG and concluded that the GPR axons terminate as a compact bundle similar to the view of GPR axons afforded in a single optical slice or a maximum projection in our study (Fig 8). However, we used z-stacks through the entire CoG depth and rotation of these stacks to examine the GPR axons in 3D. A side projection view reveals that the GPR axons did not in fact terminate in a compact bundle, but turned and projected dorsally (Fig 9A; n = 11/15). Thus, the apparent termination point identified in Fig 8 is not the actual termination of the GPR axons. In 11 preparations we could readily track the GPR axons through z-stack images and their projection into the dorsal region was apparent. For instance, applying depth coding to a maximum projection of a CoG viewed from the ventral side illustrates GPR axons traveling across the ventral surface (red) and then projecting dorsally (green) (Fig 9B). In the remaining 4/15 preparations it was not possible to detect dorsal projections of the GPR axons. Given the degree of other serotonin-IR within the CoGs, we could not resolve the GPR axons in those CoGs. However, the 11 preparations in which the GPR axons could be tracked suggest that they project dorsally toward the region in which the MCN1 neurites arborize (Fig 3). Thus, we examined the subset of preparations in which MCN1 and GPR were double labeled to determine whether MCN1 and GPR arborizations overlap.

**Fig 10:**
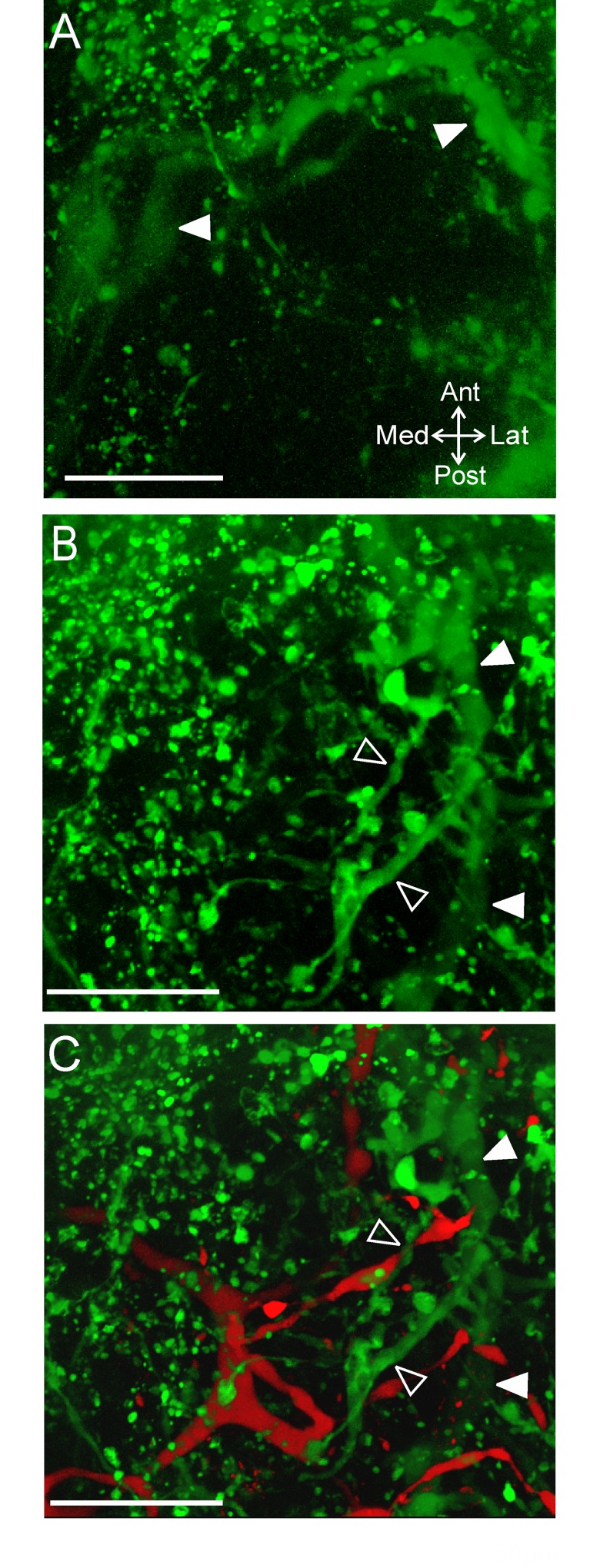
GPR projects dorsally and reaches the level of MCN1 neurites. The GPR axon bundle was traced through a series of optical slices to the level of MCN1 neurites. (A) The GPR axons (filled arrowheads) entered the CoG near the ventral surface and projected dorsally (volume rendering of z-stack, 266 slices, 0.7 μm interval). (B) GPR axons projected into the dorsal CoG as a bundle until diverging with several small branches traceable from the main bundle (open arrowheads) (volume rendering of z-stack, 196 slices, 0.7 μm interval). (C) The smaller GPR branches were in the same region of the CoG as dorsal MCN1 neurite branches (red) (196 slices, 0.7 μm interval).

By tracing the projection of the GPR axon bundle through z-stacks, we found that GPR axons overlap with MCN1 neurite branches in the dorsoventral dimension. The GPR axons remained closely associated as a bundle through the ventral CoG, but then defasciculated and arborized into finer neurites closer to the dorsal CoG surface. After the initial splaying of the individual GPR axons, it became difficult to distinguish GPR neurites from other serotonergic neuropil and the full extent of the GPR arborization could not be determined. Thus, we quantified the point in the dorsoventral dimension at which the initial defasciculation of GPR axons and branching into finer neurites occurred. We found that the point at which the GPR axons branched in the dorsoventral dimension (59.0 ± 13.2; n = 11) (Fig 9C) was consistently located at a similar depth as MCN1 smaller neurites. In double labeled preparations, we found MCN1 neurites passed near these finer GPR neurite branches (Fig 10) (n = 3/3). Previous studies indicate that the GPR termination field was much smaller than the spread of MCN1 neuropil arborization [24,39], suggesting a small region of contact from GPR to MCN1. Our findings that the GPR axons defasciculate, with multiple fine branches spreading into the neuropil instead of a small tuft of terminals [39] suggest GPR contacts onto MCN1 are more broadly distributed than initially expected.

**Fig 11:**
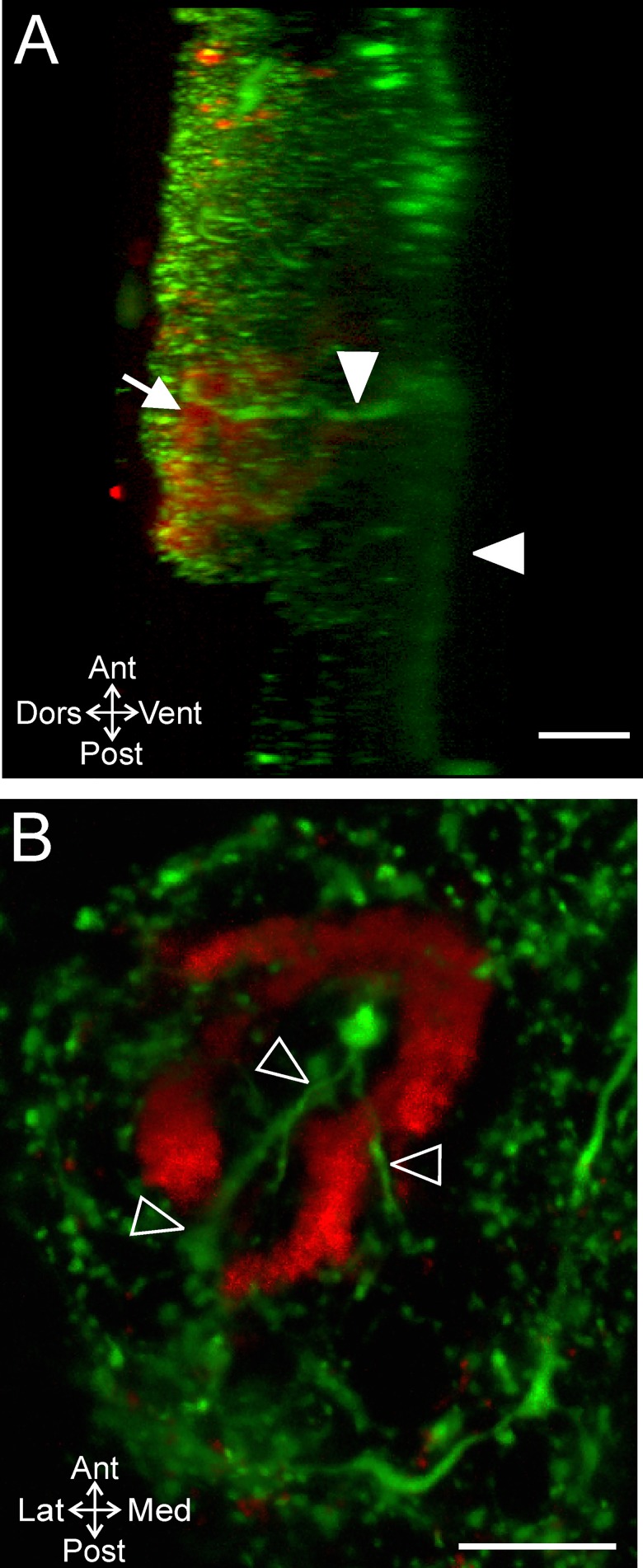
GPR projects dorsally and reaches the level of the ACO. (A) The GPR axons (filled arrowheads) entered the CoG ventrally, projected into the anterior region of the CoG, and then turned to project dorsally where the ACO (arrow) was located (268 slices, 0.8 μm interval). The CoG was imaged from the dorsal surface and the 3D rendering rotated to obtain this side view. (B) In a view from the dorsal surface of the same CoG as in (A) fine branches of the GPR axons (green: open arrowheads) after the axons defasciculated occurred in gaps in the ACO structure (red) (30 slices, 0.8 μm interval).

Given the projection of the GPR axons into the anterodorsal CoG region, we examined whether GPR projected in close proximity not only to MCN1 neurites but also to the ACO. We found that the GPR axons did indeed project near the ACO (n = 3/3). For instance, a side view of a double labeled preparation reveals the GPR axons (green) projected dorsally to the region in which the ACO (red) was located (Fig 11A). Further, GPR axons projected through gaps in the ACO structure similar to the way in which MCN1 neurites passed through gaps that are indicative of hemolymph lacunae (Fig 11B) (n = 3/3), suggesting potential convergence of ACO and GPR input to MCN1 in these regions.

### Convergence of modulatory inputs within the CoG

**Fig 12:**
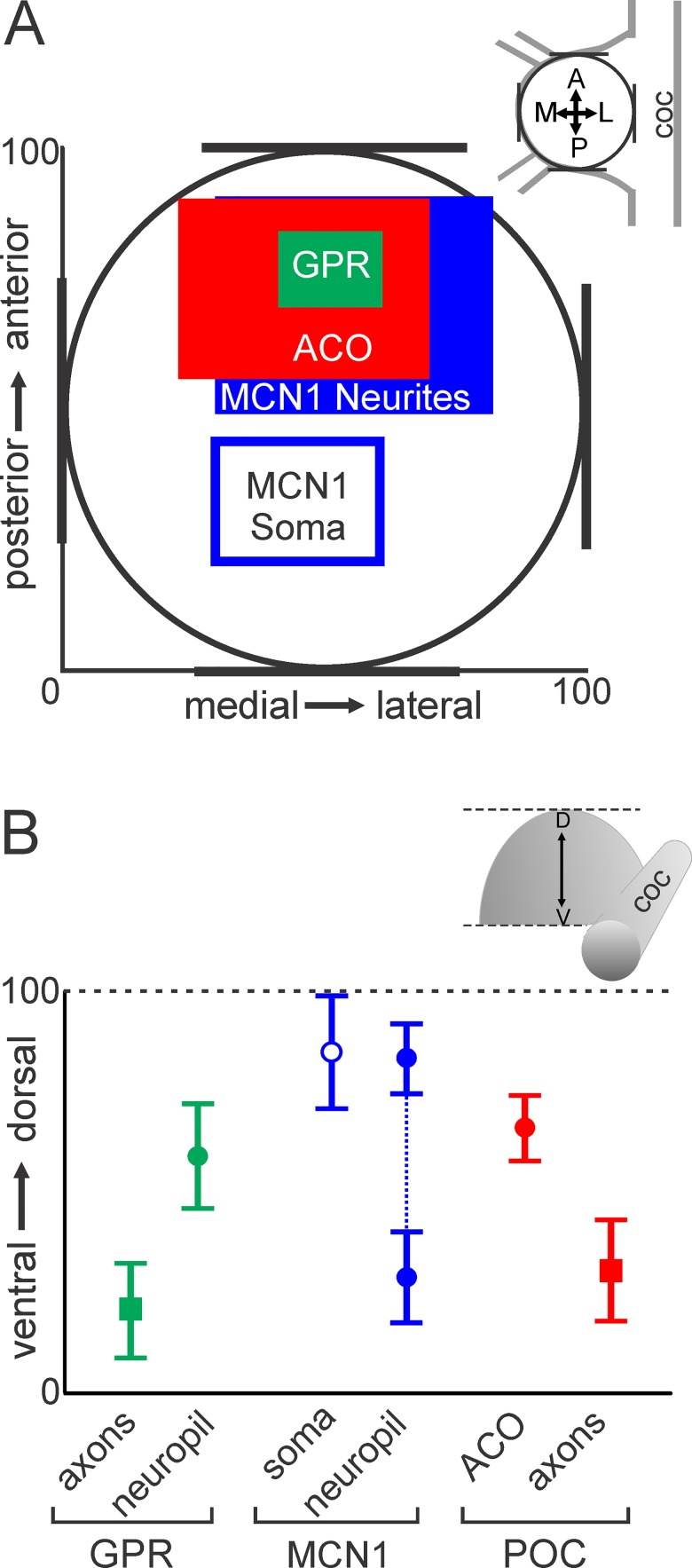
MCN1, GPR and ACO overlap in the anteroposterior, mediolateral and dorsoventral dimensions. (A) A graph of the average (± S.D.) locations in the anteroposterior and mediolateral planes for the MCN1 soma (open blue box), MCN1 neurites (filled blue box), ACO (red box) and GPR axon bundle (green box) demonstrates overlap of the MCN1 neurites with the two inputs in the anterior CoG. (B) Average locations in the dorsoventral plane demonstrate overlap of the MCN1 neurites (blue filled circles) with the ACO (red circle) and the point at which the GPR axons defasciculated (green circle) in the dorsal CoG. Additionally, the MCN1 neuropil arborization spread into the ventral CoG near the POC (red square) and GPR (green square) axons which entered the ganglion closer to the ventral surface. The MCN1 soma was the most dorsal structure (open blue circle).

The MCN1 soma was located in the posterior region of the CoG while the MCN1 neurites, ACO and GPR axon bundle were located in the anterior region (Fig 12A). Given the depth of the CoG, however, it remained possible that despite a large amount of overlap in the anteroposterior and mediolateral plane, there might be limited overlap in the dorsoventral dimension. We found that there was separation between the dorsoventral level of the MCN1 soma and dorsoventral level at which GPR or POC axons projected into the CoGs (Fig 12B; blue open circle, red and green squares). However, there was overlap in the MCN1 neurites and center of the ACO dorsoventrally (Fig 12B; red and blue filled circles). Due to the non-GPR serotonin-IR, we were not able to determine the full extent of the GPR axonal branching in the dorsoventral dimension, instead we plotted the point at which the bundle of four GPR axons defasciculated (green circle) and began arborizing into finer neuropilar processes. This measurement revealed overlap with both the MCN1 neurites and the center of the ACO in the dorsoventral plane (Fig 12B; red, blue, green filled circles). These results highlight overlap, and not segregation, of the GPR and ACO terminals relative to MCN1.

## Discussion

We examined the 3D arborizations of a modulatory projection neuron and two of its physiologically important inputs, a defined neuroendocrine organ and proprioceptive neurons. Previous anatomical studies provided information about the general location of MCN1, the ACO, and the GPR neurons in the CoG but did not include 3D or quantitative analyses [24,28,34,39], limiting the utility of the information in generating predictions of synaptic interactions. The use of 3D analysis allowed us to overcome these limitations and determine the extent to which each of the neurons extended through the dorsoventral axis of the CoG and overlapped with the others. We found that the arborizations of these neurons, which are all involved in regulating chewing and filtering rhythms, are segregated to the anterior portion of the CoGs. Within this domain, the MCN1 neurites overlap extensively with the two inputs, each relaying different information to MCN1, in the anteroposterior and mediolateral as well as the dorsoventral dimension (Fig 12). Additionally, the MCN1 neurite arborizations and ACO morphologies were coincident with each other despite qualitative structural differences, i.e., round versus elongated ACO morphology, between preparations. Further, the GPR axons were found to project farther and arborize more extensively than previously known.

### General organization

Although identified neurons in some invertebrate ganglia have stereotyped soma locations across animals, this does not seem to be the case in others [47–50]. For instance, within the STG there are preferred but not stereotypical locations of circuit neurons [49,50]. Similarly in the CoG, the MCN1 soma location varied between animals. In the STG at least one neuron has a stereotyped neuropil branching pattern, while others do not [49–51], comparable to variable branching patterns of identified neurons in other systems [48,52]. In the CoG, there was regional segregation of arborizations, but variation in their spread within that region. The extent of neuropilar segregation likely relates to functional segregation within a ganglion. For instance, there is also regional segregation of neurons in the CoG projecting to different parts of the nervous system [23,26]. In addition to neuropilar arborizations having a consistent regional localization, other consistent patterns included the GPR and POC axons reliably projecting along the ventral side of the CoG before turning and projecting dorsally, and the larger diameter MCN1 branches consistently occurring near the center of the CoG. These patterns echo organization within the STG. For instance, the axon of the sensory anterior gastric receptor (AGR) neuron projects along the STG ventral surface and projects processes dorsally into the STG to reach arborizations of local circuit neurons. Additionally, larger diameter branches of STG neurons occur in its center similar to the organization of the MCN1 branches we found in the CoG [46,53–56]. Both the ventral entry of axons and the large primary neurites occurring centrally suggests the possibility of similar developmental constraints restricting different cell compartments to particular regions in the CoG and STG.

### Consistency of neuroendocrine organ and projection neuron arborizations

Neuroendocrine cells which utilize hormonal signaling through the circulation also act more locally via paracrine signaling [35–37]. For instance, hypothalamic cells that use oxytocin for hormonal reproductive functions also have oxytocin-mediated paracrine actions within amygdala fear circuitry [37]. In many cases, the targets of paracrine actions are populations of neurons, making it difficult to determine the extent of overlap between individual neurons and the paracrine input. In the STNS, we took advantage of the neuroendocrine ACO targeting projection neurons including MCN1, which occurs as a single copy in each CoG. ACO uses CabTRP Ia paracrine actions to influence MCN1. Brief (30 s) POC axon stimulation triggers a long-lasting MCN1 activation (~20 min) [28]. Given the potential for peptidergic volume transmission, it was possible that the ACO neuroendocrine organ and the MCN1 arborizations would share general regional overlap but not tightly coordinated overlap of their arbors. However, we found that the MCN1 neuropilar arborization closely co-localized with ACO morphology, which varied across preparations from a round to an elongated shape. Further, MCN1 processes wrapped through and around gaps in the ACO.

It remains unknown whether the unexpected strong anatomical correlation of MCN1 and ACO processes reflects structural constraints such as vasculature that dictate neurite locations, or has functional implications. For instance, transmission may be more spatially restricted than the broad volume transmission expected for a modulatory peptide transmitter or degradation mechanisms might restrict peptide diffusion sufficiently to require closer association of the ACO and MCN1 neurites [57,58]. The spatial similarity and intertwined nature of these neurons may be necessary for ACO released CabTRP Ia to reach a large pool of available MCN1 CabTRP Ia receptors enabling the long-lasting strong MCN1 activation by the ACO [28,59]. Further, despite paracrine release having the potential to act on many nearby receptors, peptidases can limit released peptide actions [57,60–62]. In particular ACO released CabTRP Ia actions on MCN1 persist for a longer duration in the presence of a peptidase inhibitor [28]. This could reflect CabTRP Ia acting on the same set of receptors for longer durations, or CabTRP Ia reaching additional receptors on MCN1 in the absence of peptidase activity. Thus, despite the co-variance in ACO and MCN1 arborizations, we do not yet know the extent to which this may be necessary for ACO-released peptide to reach sufficient MCN1 receptors without being degraded. A more in depth understanding of this peptidergic signaling will require localization of peptide receptors and membrane bound peptidases [57,63]. Identification of invertebrate peptide receptors lags behind that of peptides, however, great strides are being made in identifying neuropeptide G protein-coupled receptors including recent identification of a peptide receptor in the crustacean STNS [57,64].

### Sensory neuron arborization

Although most information regarding proprioceptive regulation of motor output involves actions at the circuit level, proprioceptive feedback also regulates the activity of projection neurons. Monosynaptic connections such as the GPR, AGR, and posterior stomach receptor (PSR) cells onto MCN1 and other projection neurons occur in the STNS [12,27,65]. In other systems the connectivity remains unexplored or is more likely to be polysynaptic, such as the pathways targeting reticulospinal neurons in vertebrate locomotion [13,66–67]. In all of these systems, little detailed anatomical information is available about these connections.

The proprioceptor GPR neurons project across the ventral surface of the CoG as a bundle of closely associated axons. Previous examination suggested that the GPR axons terminated as a compact bundle, abruptly ending with a small arborization of fine processes, suggestive of sparse connectivity onto MCN1 [39]. We now find that the GPR axons project beyond this apparent compact termination and their arborization is more extensive than previously thought. The use of 3D analysis and a mounting technique that prevents compression of the CoG likely account for the different perspective on the GPR terminations in this study. The GPR axons remained closely associated as they projected dorsally, before defasciculating and arborizing in the region of both ACO processes and small MCN1 neurites. This indicates the potential for a more distributed GPR input onto MCN1 than previously suggested; potentially enabling this proprioceptive feedback to more effectively override effects of other inputs, or to spatially co-localize with more inputs.

While specific interactions between the ACO and GPR in this system have not been identified, there are interactions between multiple extrinsic inputs targeting projection neurons [65,68,69]. For instance, activation of the PSR cells presynaptically inhibits release from the AGR sensory neuron onto one projection neuron target, while enhancing AGR release onto another [65]. Similarly, stimulation of the mechanosensory ventral cardiac neurons (VCNs) gates out GPR effects on MCN1 [68]. The close association of the GPR axons as a bundle through much of the CoG may allow other inputs to readily target the entire GPR neuron population and gate their CoG actions. Ionotropic and metabotropic receptors occur on axons even some distance from their terminals [70,71]. This includes for instance, GABAergic synapses onto the axon trunk of jaw muscle spindle afferents blocking action potential propagation into one compartment of these sensory axons [70]. Thus, it is possible that the bundle of GPR axons could be targeted prior to their defasciculating and arborizing. This could allow a block of all GPR actions within the CoG, leaving only its actions on the motor circuits in the STG. Alternatively, given the arborization of fine branches of GPR axons in the dorsal CoG, inputs might selectively gate effects mediated by a subset of the GPR axon terminals while leaving other GPR synapses within the CoG effective. Such fine-tuning is evident at the STG terminals of MCN1 where GPR presynaptically inhibits release of a subset of the MCN1 cotransmitters [72].

## Conclusions

Integration of information from multiple pathways targeting projection neurons is necessary for the selection of appropriate behaviors [13,14,16]. Here we were able to examine the relative 3D structures of multiple identified inputs that converge onto MCN1 with high resolution since it occurs as a single copy rather than a group of multiple cells. This enabled us to identify similarities in MCN1 morphology and the ACO neuroendocrine structure without ambiguity that can exist when examining a neuronal population. The extensive overlap of the ACO and MCN1 neuropil, and the larger ramification of GPR than anticipated supports a dispersed overlapping organization of these two defined inputs relative to their projection neuron target, providing a possible substrate for local interactions to determine their convergent regulation of MCN1 activity.

## Supporting Information

S1 Dataset Raw data for Figs 2–5, 8, 9 and 12 and data in text only. (XLSX)
